# The correlation between periodontitis and fatty liver and the improvement of NAFLD by periodontal treatment

**DOI:** 10.3389/froh.2026.1706821

**Published:** 2026-06-08

**Authors:** Meiying Xiang, Yusi Hua

**Affiliations:** Department of Anaesthesiology, West China Hospital, Sichuan University, Chengdu, China

**Keywords:** NAFLD, oral-gut-liver axis, periodontitis, *Porphyromonas gingivalis*, systemic inflammation

## Abstract

**Background:**

Emerging evidence highlights a pathophysiological interplay between periodontitis and non-alcoholic fatty liver disease yet the mechanistic underpinnings and therapeutic implications remain contentious. This review systematically elucidates molecular crosstalk through the “oral-gut-liver axis” and “oral-liver axis”.

**Methods:**

A comprehensive literature review was conducted using PubMed, Scopus and Web of Science, employing keywords related to periodontal disease and non-alcoholic fatty liver disease.

**Results:**

Analysis of 16 original studies revealed that periodontitis and its associated pathogens promote the progression of non-alcoholic fatty liver disease through multiple pathways: (1) activation of hepatic inflammatory responses (elevated IL-6, IL-17, and TNF-α levels), (2) exacerbation of metabolic dysregulation (increased HOMA-IR, ALT, and AST), and (3) disruption of the oral-gut-liver axis. Notably, non-surgical periodontal therapy demonstrated therapeutic potential by simultaneously improving periodontal health and attenuating non-alcoholic fatty liver disease progression through reduction of hepatic pro-inflammatory cytokines and fibrogenic mediators.

**Conclusions:**

Periodontitis may exacerbate systemic inflammation via the oral-liver and oral-gut-liver axes, inducing insulin resistance and promoting non-alcoholic fatty liver disease. Non-surgical periodontal therapy can improve non-alcoholic fatty liver disease, but methodological heterogeneity in current studies necessitates further prospective research to clarify their relationship.

## 1 Introduction

Periodontitis is a chronic inflammatory disease affecting the tooth-supporting structures, characterized by the immune-mediated destruction of alveolar bone and gingival soft tissues in response to bacterial dental plaque, leading to various oral health complications (1). Notably, the health implications of periodontitis extend beyond the oral cavity, as it has been associated with numerous systemic conditions (2). In addition to established links with cardiovascular disease (3), renal disease (4), and mental health disorders (5), a growing body of evidence from both animal and human studies suggests a potential association between periodontitis and non-alcoholic fatty liver disease (NAFLD).

**Figure 1:**
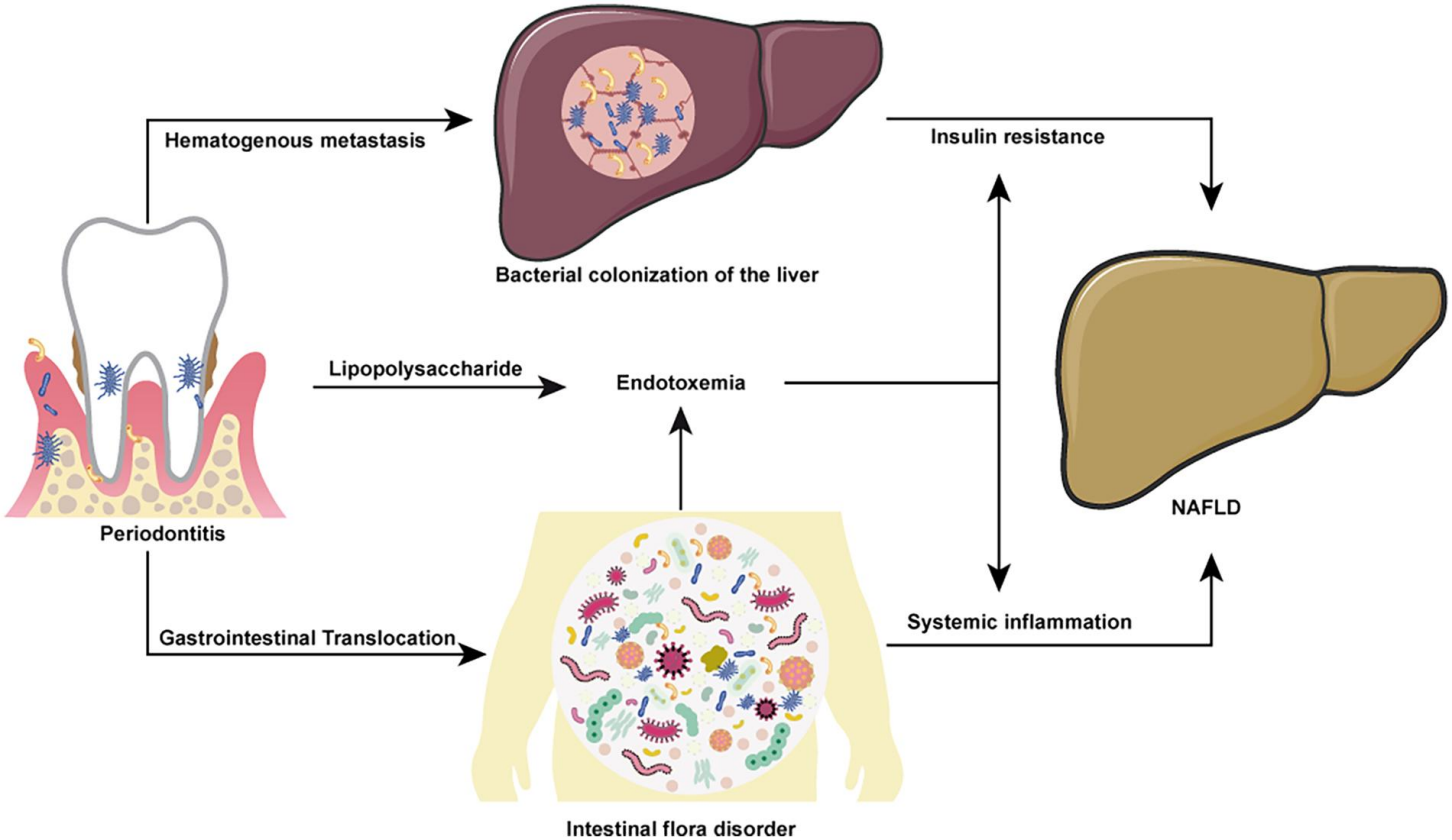
The correlation between periodontitis and NAFLD. Periodontitis and its associated pathogens (e.g., Porphyromonas gingivalis) may facilitate the onset and progression of NAFLD via the “Oral-Gut-Liver Axis” and “Oral-Liver Axis”. Key mechanisms involve bacterial and endotoxin dissemination, gut microbiota dysbiosis, intestinal barrier dysfunction, systemic inflammatory activation, and the development of insulin resistance, collectively culminating in hepatic steatosis and hepatocellular injury. NAFLD, Non-alcoholic fatty liver disease.

NAFLD, as defined by the American Association for the Study of Liver Disease (AASLD), develops in the absence of significant alcohol consumption (typically defined as less than approximately 20 g/day for women and 30 g/day for men) (6). Characterized by excessive fat accumulation in the liver, NAFLD is commonly associated with obesity, diabetes, and metabolic syndrome, but it can also affect non-obese individuals. Recently, the term MAFLD (Metabolic Dysfunction-Associated Fatty Liver Disease) has been proposed to emphasize metabolic dysfunction; however, NAFLD remains widely used in the literature. The key markers of NAFLD include elevated hepatic transaminases (ALT, AST), oxidative stress indicators, and liver biopsy as the diagnostic gold standard. The disease spectrum encompasses simple steatosis with or without inflammation (non-alcoholic fatty liver, NAFL), progressing to steatohepatitis with inflammation and hepatocellular injury (non-alcoholic steatohepatitis, NASH), fibrosis, and ultimately cirrhosis (7). Globally, NAFLD affects an estimated 25%–30% of the population, with higher prevalence rates observed in Asia followed by Europe and North America. In China, the prevalence has reached approximately 29% (8). NAFLD represents a significant cause of morbidity and mortality, yet specific and highly effective therapeutic interventions remain limited. Its pathogenesis is highly complex. The traditional “two-hit” hypothesis centered on insulin resistance as the key driver. More recently, the “multiple parallel hits” theory has emerged, proposing that factors such as gut microbiota dysbiosis, epigenetic modifications, and environmental influences also play crucial roles in the development and progression of NAFLD (9). Accumulating evidence from diverse fields indicates that the oral inflammation and pathological shifts in the oral microbiome triggered by periodontitis are closely linked to the onset and progression of NAFLD (10) (Figure 1).

A comprehensive scoping review of the literature was conducted to identify relevant studies exploring the association between periodontitis and non-alcoholic fatty liver disease (NAFLD)/metabolic dysfunction-associated fatty liver disease (MAFLD). The electronic databases PubMed, Scopus, and Web of Science were systematically searched using a combination of controlled vocabulary (MeSH/Emtree) and free-text terms related to periodontitis (e.g., “periodontal disease”, “periodontitis”, “gingivitis”) and fatty liver disease (e.g., “non-alcoholic fatty liver disease”, “NAFLD”, “MAFLD”, “steatohepatitis”). The search strategy was designed and executed with Boolean operators (AND/OR) to capture all relevant publications. Inclusion criteria were: (1) original research articles (observational studies, clinical trials, or experimental studies in animals); (2) studies explicitly evaluating periodontitis or periodontal pathogens as an exposure or intervention; (3) studies reporting NAFLD/MAFLD outcomes (e.g., biochemical, histological, imaging-based, or clinical diagnostic criteria). Exclusion criteria included: (1) non-English publications; (2) review articles, editorials, case reports, or conference abstracts without original data; (3) studies focusing solely on alcoholic liver disease or other hepatic pathologies unrelated to NAFLD/MAFLD.

Numerous epidemiological and clinical studies have investigated the relationship between periodontitis and NAFLD. However, findings regarding this potential association remain inconclusive and sometimes contradictory, likely due to variations in study design. Therefore, this review aims to synthesize evidence from diverse study types to elucidate the specific association between periodontal disease and NAFLD. Furthermore, by examining the impact of non-surgical periodontal therapy on NAFLD improvement, this review seeks to provide insights that could inform novel therapeutic perspectives for NAFLD management.

## 2 Periodontitis, periodontal pathogens, and NAFLD

### 2.1 Clinical association between periodontitis and NAFLD: current evidence and controversies

The existence of a direct association between periodontitis and NAFLD remains controversial. NAFLD and metabolic syndrome (encompassing obesity, hypertension, and diabetes) exhibit bidirectional influences (11). Dysregulated glucose and lipid metabolism, oxidative stress, and chronic inflammation are key pathogenic drivers of NAFLD (12). Furthermore, the role of altered host microbiota in the pathogenesis of NAFLD has gained increasing attention (13).

Periodontitis is associated with elevated levels of numerous systemic inflammatory markers (14), which are recognized as potential risk factors for various diseases, including NAFLD. Intriguingly, current research investigating a specific association between periodontitis and NAFLD has yielded conflicting results. This inconsistency may stem from variations in the diagnostic criteria for periodontitis employed across different studies. These criteria generally fall into two categories: Clinical Parameters: Including one or more of the following: clinical attachment level (CAL), probing depth (PD), bleeding on probing (BOP), Community Periodontal Index (CPI), or tooth loss; microbiological Parameters: Including IgG antibody titers against specific periodontal pathogens [e.g., *Aggregatibacter actinomycetemcomitans* (*A. actinomycetemcomitans*), *Fusobacterium nucleatum* (*F. nucleatum*), and *Porphyromonas gingivalis* (*P. gingivalis*) (15, 16)], IgG antibody titers specific to *P. gingivalis*, or the isolation of *P. gingivalis*. Bacterial load measurements reflect pathogen translocation rather than serving as diagnostic criteria for periodontitis.

Among observational studies (cross-sectional, case-control, and cohort), nearly all studies, with one exception (17), reported a significant association between periodontitis (defined by clinical and/or microbiological parameters) and NAFLD (14–24). However, two meta-analyses that addressed significant heterogeneity among included studies also produced conflicting conclusions regarding the periodontitis-NAFLD link (25, 26). One meta-analysis, incorporating 7 studies that exclusively used clinical parameters to define periodontitis, concluded that “current evidence fails to establish a link between periodontitis and NAFLD” (25). Notably, the other meta-analysis reported: “The investigation revealed a pronounced correlation between periodontitis and NAFLD. Nonetheless, upon adjusting for multiple metabolic parameters, this association was attenuated, indicating that these metabolic aberrations, rather than periodontitis perse, might serve as predisposing elements for NAFLD.” (26). However, as this meta-analysis did not conduct further analyses to identify the specific metabolic confounders responsible for the attenuation of the association, the exact nature of these modifying factors remains unclear from its findings. To provide a comprehensive overview of the existing evidence investigating the link between periodontitis and NAFLD, we have summarized key studies in Tables 1, 2. The table synthesizes evidence ranging from animal models to human observational studies, detailing the study design, periodontal and NAFLD diagnostic parameters used, and primary outcomes (31–40). The compilation facilitates an examination of the breadth of research approaches and findings in this field.

**Table 1:** Clinical studies on the correlation between periodontitis and fatty liver.

Study	Study type	Counts	Periodontal parameters	NAFLD diagnosis parameters	Main outcome
Akinkugbe et al. (16)	Population based cohort investigation	2,623	CAL ≥3 mm;	Abdominal sonography;	History of periodontitis may be a risk factor for NAFLD.
			PD ≥4 mm.	ALT	
Iwasaki et al. (21)	Cross-sectional study	1,226	PPD ≥ 4 mm	Abdominal ultrasonography	Having PPD ≥ 4 mm may be a risk factor for ultrasound-diagnosed NAFLD
Helenius Hietala et al. (27)	Population-based cohort study	6,165	Periodontal pocket ≥4 mm deep	Fatty liver index	Periodontal disease might present a modifiable risk factor for chronic liver disease
Shin (28)	Nationwide cross-sectional study	4,061	Community periodontal index; periodontal pockets	FLI and HSI	Presence of periodontal pockets may be independently associated with NAFLD indicators.
Sato et al. (29)	Cross-sectional study	164	*P. gingivali*s positivity;	VCTE and magnetic resonance imaging	Individuals with > 10 periodontal pockets of depths ≥ 4 mm demonstrated significantly increased hepatic stiffness.
			periodontal pockets		
Bordagaray et al. (30)	Cross-sectional study	59	Number, mean diameter and periapical index of the apical lesions of endodontic origin	TNF-α, IL-4, IL-9, IL-10, IL-17A and IL-22;	Apical periodontitis is associated with higher serum hepatic transaminases ALT and AST.
				ALT and AST	

CAL: Clinical attachment level, PD: Probing depth, ALT: Alanine aminotransferase, NAFLD: Non-alcoholic fatty liver disease, PPD: Probing pocket depth, FLI: Fatty liver index, HSI: Hepatic steatosis index, VCTE: Vibration-controlled transient elastography, TNF-α: Tumor necrosis factor-alpha, IL-4: Interleukin-4, IL-9: Interleukin-9, IL-10: Interleukin-10, IL-22: Interleukin-22, HCC: Hepatocellular carcinoma, AST: Aspartate aminotransferase.

**Table 2:** Animal model studies on the correlation between periodontitis and fatty liver.

Study	Counts	Periodontal parameters	NAFLD diagnosis parameters	Main outcome
Liu et al. (31)	10	*P. gingivalis*;	Lee's index and liver index	Inflammatory response of liver in mice induced by orally administered Porphyromonas gingivalis
		Oral administration	ALT, AST, and TG	
			IL-17a, IL-6, and ROR-*γ*t	
Kuraji et al. (32)	24	Ligature placement, and *P. gingivalis* oral infection	ALT, AST;	Experimental periodontitis induced by *P. gingivalis* led to the progression of NASH in rats with fatty liver.
			Endotoxin and C-reactive protein	
Fujita et al. (33)	28	LPS from *P. gingivalis* infection	Lipid deposition and infiltrating inflammatory;	*P. gingivalis*-LPS from the oral cavity to the liver plays an important role in disease exacerbation of NASH.
			NAS score	
Ni et al. (34)	38	Performing periodontal ligation with *P. gingivalis*	HOMA-IR, OGTT, FFA	Periodontitis increases insulin resistance while scaling and root planning could improve insulin resistance.
Pessoa et al. (35)	18	One or two ligatures	Steatosis score; GSH, MDA, MPO, cholesterol and triglycerides ALT and AST	One or two ligatures inducing periodontitis were both sufficient to cause fatty liver
Sasaki et al. (36)	20	Intravenous sonicated *P. gingivalis*	Glucose/lipid metabolism, liver steatosis, and gut microbiota	Blood infusion of *P. gingivalis* contributes to NAFLD and alters the gut microbiota
Xing et al. (37)	12	Tooth ligation;	TNF-α, IL-1β, TG, AST, ALT	Tooth ligation with silk thread induces a liver inflammatory response and steatosis in rats.
		Alveolar bone loss		
Yao et al. (38)	24	IL-17 and IL-1β;	Hepatic steatosis;	*P. gingivalis* is a risk factor for the development of nonalcoholic fatty liver disease via ferroptosis
		Alveolar bone loss	Inflammatory cells infiltrated;	
			ALT and AST	
Ding et al. (39)	16	amount of bone loss;	TG, IL-1β and TNF-α;	Exosomes associated with periodontitis promote hepatocyte adipogenesis
		greater alveolar crest resorption and a greater distance	NAFLD activity score	
Ohno et al. (40)	18	inflammatory cell infiltration of gingiva, alveolar bone resorption	HCC progression, and expression of genes encoding inflammatory cytokines	HCC progression was greater in STAM mice with experimental periodontitis

ALT: Alanine aminotransferase, AST: Aspartate aminotransferase, TG: Triglycerides, IL-17A: Interleukin-17A, IL-6: Interleukin-6, ROR-γt: Retinoic acid receptor-related orphan receptor gamma t, NASH: Non-alcoholic steatohepatitis, LPS: Lipopolysaccharide, NAS score: Nonalcoholic steatohepatitis activity score, HOMA-IR: Homeostatic model assessment of insulin resistance, OGTT: Oral glucose tolerance test, FFA: Free fatty acids, GSH: Glutathione, MDA: Malondialdehyde, MPO: Myeloperoxidase, TNF-α: Tumor necrosis factor-alpha, IL-1β: Interleukin-1 beta, HCC: Hepatocellular carcinoma.

Collectively, this evidence indicates that the question of a direct association between periodontitis and NAFLD remains unresolved and controversial. Comprehensive, systematic, large-scale clinical studies or meta-analyses with minimal heterogeneity are warranted to definitively address this ongoing debate among researchers worldwide.

While the majority of observational studies report a positive association between periodontitis and NAFLD, the strength and independence of this link remain contested. Although heterogeneity in periodontal diagnostic criteria—ranging from clinical parameters (e.g., probing depth, clinical attachment loss) to serological markers of pathogen exposure—has been widely cited as a source of inconsistency (25, 26), this explanation alone is insufficient. A more nuanced critique must consider how differences in study populations, sample sizes, and, most critically, the handling of metabolic confounders in statistical analyses may fundamentally alter the observed relationship.

For instance, several large cross-sectional studies conducted in East Asian populations (e.g., Japan and Korea) consistently report significant associations even after adjusting for BMI, diabetes, and dyslipidemia (21, 28, 29). In contrast, the meta-analysis by Xu et al. (25), which concluded no robust link, pooled studies that either lacked adjustment for key metabolic variables or relied solely on self-reported comorbidities without objective biomarkers. This raises the possibility that unmeasured or inadequately controlled metabolic dysfunction—not periodontitis *per se*—may drive both conditions, creating a spurious association when confounding is not rigorously addressed.

Moreover, ethnic and genetic background may modulate susceptibility to both periodontitis and NAFLD. Studies in Hispanic and European cohorts have shown varying effect sizes (16, 17), potentially reflecting differences in inflammatory response profiles, gut microbiome composition, or lifestyle factors. Small sample sizes in some animal or pilot human studies further limit statistical power to detect modest but biologically relevant effects, especially when stratifying by metabolic status.

Crucially, the attenuation of the periodontitis–NAFLD association after multivariate adjustment for metabolic syndrome components—as noted in the meta-analysis by Wijarnpreecha et al. (26)—suggests that periodontitis may act less as an independent risk factor and more as a marker or amplifier of underlying metabolic dysregulation. However, without individual participant data or standardized covariate adjustment across studies, it remains unclear whether periodontitis contributes causally to NAFLD pathogenesis or merely coexists within a shared inflammatory-metabolic milieu.

### 2.2 Mechanisms of periodontal pathogen-induced NAFLD: the oral-gut-liver and oral-liver axes

**Figure 2:**
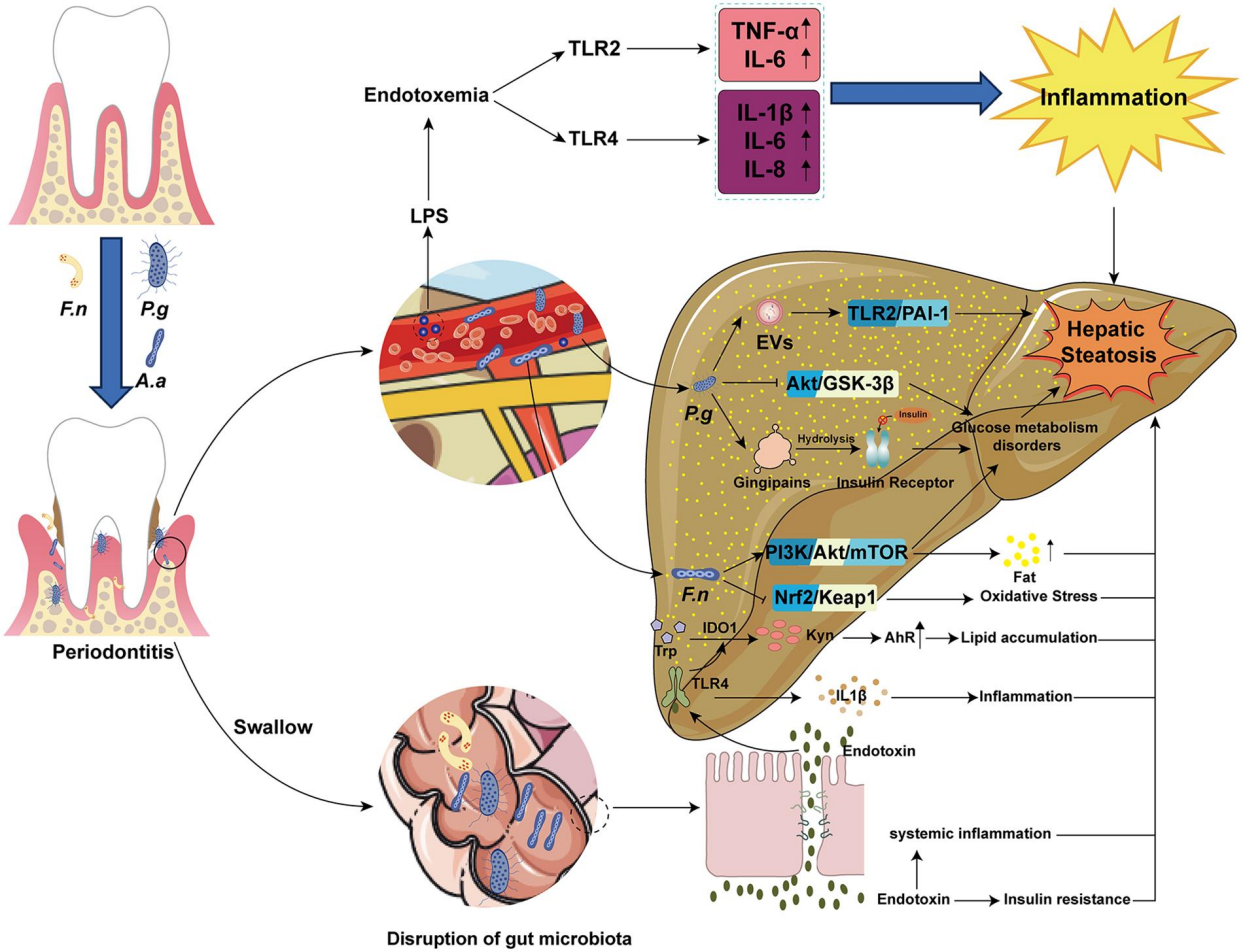
Periodontitis pathogenic bacteria cause NFALD through the oral–liver axis and the oral–gut–liver axis. Schematic representation of the bidirectional links between periodontitis and non-alcoholic fatty liver disease (NAFLD) via the oral–liver and oral–gut–liver axes. In periodontitis, pathogenic bacteria such as *F.n*, *P.g*, and *A.a* colonize the periodontal pocket, leading to local inflammation and tissue destruction. These bacteria or their virulence factors (e.g., LPS, gingipains, EVs) enter systemic circulation through bacteremia, activating Toll-like receptors (TLR2 and TLR4) in hepatocytes and immune cells, resulting in increased pro-inflammatory cytokines (TNF-α, IL-6, IL-1β, IL-8) and systemic inflammation. Directly, *P.g* can impair insulin signaling via Akt/GSK-3β pathway disruption and promote hepatic steatosis through PI3K/Akt/mTOR activation. Additionally, swallowed oral microbes contribute to gut microbiota dysbiosis, compromising intestinal barrier integrity and increasing endotoxin (e.g., LPS) translocation into the portal circulation. This triggers hepatic inflammation via TLR4 signaling, oxidative stress, lipid accumulation (via AhR activation by kynurenine), and insulin resistance. The interplay between oral pathogens, gut dysbiosis, and hepatic metabolic dysfunction highlights a critical role for periodontal health in NAFLD development and progression. *F.n*, *Fusobacterium nucleatum*; *P.g*, *Porphyromonas gingivalis*; *A.a*, *Aggregatibacter actinomycetemcomitans*; AhR, Aryl hydrocarbon receptor; Akt, Protein kinase B; EVs, Extracellular vesicles; LPS, Lipopolysaccharide; IL-1β, Interleukin-1 beta; IL-6, Interleukin-6; IL-8, Interleukin-8; GSK-3β, Glycogen synthase kinase-3 beta; mTOR, Mammalian target of rapamycin; Nrf2/Keap1, Nuclear factor erythroid 2–related factor 2/Kelch-like ECH-associated protein 1; PI3K, Phosphoinositide 3-kinase; TLR2/TLR4, Toll-like receptor 2/Toll-like receptor 4; TNF-α, Tumor necrosis factor-alpha.

Research concerning periodontal pathogens associated with NAFLD has predominantly centered on three principal periodontopathic bacteria: *A. actinomycetemcomitans*, *F. nucleatum*, and *P. gingivalis*. Among clinical studies examining the link between periodontal disease and NAFLD, four investigations (15, 16, 21, 41) utilizing microbiological parameters for periodontal disease diagnosis identified a significant association between the presence of periodontal pathogens, notably *P. gingivalis*, and both the onset and progression of NAFLD. Current consensus posits that periodontal pathogens contribute to NAFLD pathogenesis primarily via two distinct pathways: the oral-liver axis and the oral-gut-liver axis. The core mechanism underlying the oral-liver axis is systemic inflammation, whereas the oral-gut-liver axis centers on gut microbial dysbiosis (clinical, or presumptive) (Figure 2).

#### 2.2.1 The oral-liver axis

The oral-liver axis refers to a cross-organ communication network established between the oral microbiota, their metabolites, and the liver. Core mechanisms underlying this axis involve microbial translocation, systemic inflammation, and metabolic reprogramming. Patients with periodontitis exhibit a 1.5- to 2.0-fold increased prevalence of NAFLD compared to healthy individuals. Concurrently, the abundance of *P. gingivalis* is significantly elevated in the oral cavity of NAFLD patients (20), suggesting a potential bidirectional relationship between periodontitis and NAFLD. Periodontitis is characterized by gingival ulceration and destruction of connective tissue, leading to the formation of deep periodontal pockets. These pathological changes create a direct conduit for periodontal pathogens and their metabolites to enter the systemic circulation. Once disseminated, bacterial components such as fimbriae, lipopolysaccharide (LPS), and proteases can reach the liver. This triggers alterations in key signaling pathways and factors, resulting in hepatocyte injury. Ultimately, this cascade leads to dysregulation of glucose and lipid metabolism, insulin resistance, and hepatic fibrosis (experimental, clinical).

*P. gingivalis*, a major periodontal pathogen, represents a significant risk factor for NAFLD progression. Specifically, antibody titers against *P. gingivalis* possessing type 4 fimbriae (FimA) show a significant correlation with fibrosis progression (16). In an experimental model using SNAP26b-labeled *P. gingivalis* to induce periodontitis in mice, *P. gingivalis* was detected remotely colonizing the liver. In an experimental model *in vivo*, periodontitis was induced in mice by oral inoculation of SNAP26b-labeled Porphyromonas gingivalis (strain ATCC 33277) administered via oral gavage three times per week for 8 weeks. Pg colonization in the liver was detected using fluorescence imaging, indicating systemic translocation from the oral cavity. Elevated blood glucose levels promoted this translocation, likely attributable to hyperglycemia-induced vascular endothelial damage (42). *P. gingivalis*-derived gingipains increase vascular permeability and inhibit leukocyte transendothelial migration, providing a mechanistic basis for the pathogen's distal colonization (43). Hepatic colonization by *P. gingivalis* directly inhibits the Akt/GSK-3β signaling pathway and the phosphorylation of glycogen synthase, reducing glycogen synthesis. *P. gingivalis* also indirectly impairs hepatic glycogen synthesis via upregulation of tumor necrosis factor-alpha (TNF-α) (42). Beyond direct colonization, *P. gingivalis*-derived endotoxins, such as LPS, enter the circulation, inducing systemic endotoxemia. This accelerates hepatic steatosis and fibrosis. Circulating LPS activates Toll-like receptors (TLRs): TLR2 activation increases macrophage production of cytokines like TNF-α and interleukin-6 (IL-6), while TLR4 activation elevates levels of interleukin-1β (IL-1β), IL-6, and interleukin-8 (IL-8). LPS also contributes to intracellular lipid accumulation and inflammation via nuclear factor kappa B (NF-*κ*B) and c-Jun NH2-terminal kinase (JNK) signaling (44, 45). Recent evidence indicates that *P. gingivalis*-derived extracellular vesicles (EVs) activate the PI3K/Akt/NF-*κ*B pathway via the TLR2/plasminogen activator inhibitor-1 (PAI-1) axis, upregulating hepatic PAI-1 expression. Proteases produced by *P. gingivalis* directly hydrolyze the functional insulin-binding domain of the insulin receptor (INSR), impairing insulin binding and inducing insulin resistance (46). Furthermore, these proteases activate hepatic stellate cells (HSCs) via protease-activated receptor 2 (PAR-2) and transforming growth factor-beta (TGF-*β*) pathways, promoting hepatic fibrosis (47).

*F. nucleatum* exhibits coaggregation properties, facilitating the transport of other periodontal bacteria (48). *F. nucleatum* influences hepatic lipid synthesis, leading to hyperlipidemia; potentially exacerbating the progression of atherosclerosis, a condition linked to periodontitis. Upon hepatic colonization, *F. nucleatum* triggers the PI3K/Akt/mTOR signaling cascade, resulting in increased glycolytic activity and enhanced lipogenesis in hepatocytes (49). While these findings support a causal link between PD and atherosclerosis (50, 51), research by Peiyao Wu et al. specifically demonstrated that oral inoculation of *F. nucleatum* in apolipoprotein E knockout (ApoE^−/−^) mice increased body weight, liver weight, and elevated levels of pro-inflammatory cytokines and lipids in both serum and liver. A core mechanism identified was *F. nucleatum*-mediated suppression of the nuclear factor erythroid 2–related factor 2/Kelch-like ECH-associated protein 1 (Nrf2/Keap1) antioxidant pathway, promoting hepatic steatosis (52).

#### 2.2.2 The oral-gut-liver axis

The oral-gut-liver axis denotes the intricate bidirectional communication network connecting the oral cavity, gastrointestinal tract, and liver. This network is mediated by microbiota, their metabolites, and immune-inflammatory signaling pathways (47). Periodontitis is frequently associated with oral dysbiosis, typified by an overabundance of keystone pathogens (e.g., *A. actinomycetemcomitans*, *F. nucleatum*, *P. gingivalis*) and diminished microbial diversity. Pathobionts from the periodontal niche can translocate to the gut via swallowing, contributing to gut dysbiosis. This dysbiosis compromises intestinal barrier integrity by disrupting epithelial tight junctions, thereby increasing intestinal permeability. Consequently, bacterial metabolites, including endotoxins (e.g., LPS), gain access to the portal circulation. This translocation can drive systemic metabolic dysregulation, hepatic inflammation, and steatosis (lipid accumulation). Importantly, periodontitis and systemic metabolic disorders may act synergistically, perpetuating dysbiosis in both the oral and gut microbiomes, which in turn promotes insulin resistance and chronic systemic inflammation (53). Supporting this axis, Min Wang et al. demonstrated that gavage of salivary microbiota derived from periodontitis patients into obese mice perturbed the gut microbiota composition and induced intestinal barrier dysfunction. This disruption facilitated increased LPS translocation to the liver, resulting in upregulation of TLR4, IL-1β, IDO1, and AhR expression. Consequently, these changes exacerbated hepatic inflammation and lipid deposition (54). Accumulating evidence from animal models indicates that P. gingivalis infection elicits disturbances in glucose and lipid homeostasis, hepatic inflammation, and intestinal dysbiosis. These effects represent a plausible mechanism underlying *P. gingivalis*-mediated NAFLD pathogenesis (55). Specifically, oral challenge with Porphyromonas gingivalis significantly altered the murine gut microbiota profile, characterized by an increased Bacteroidetes/Firmicutes ratio. Furthermore, elevated levels of bacterial DNA were detected in the livers of infected mice, accompanied by increased serum endotoxin and systemic inflammation. Critically, alterations in the gut microbiota preceded the manifestation of systemic inflammatory responses (experimental, or presumptive) (56).

## 3 The impact of periodontitis treatment on the progression of NAFLD

**Figure 3:**
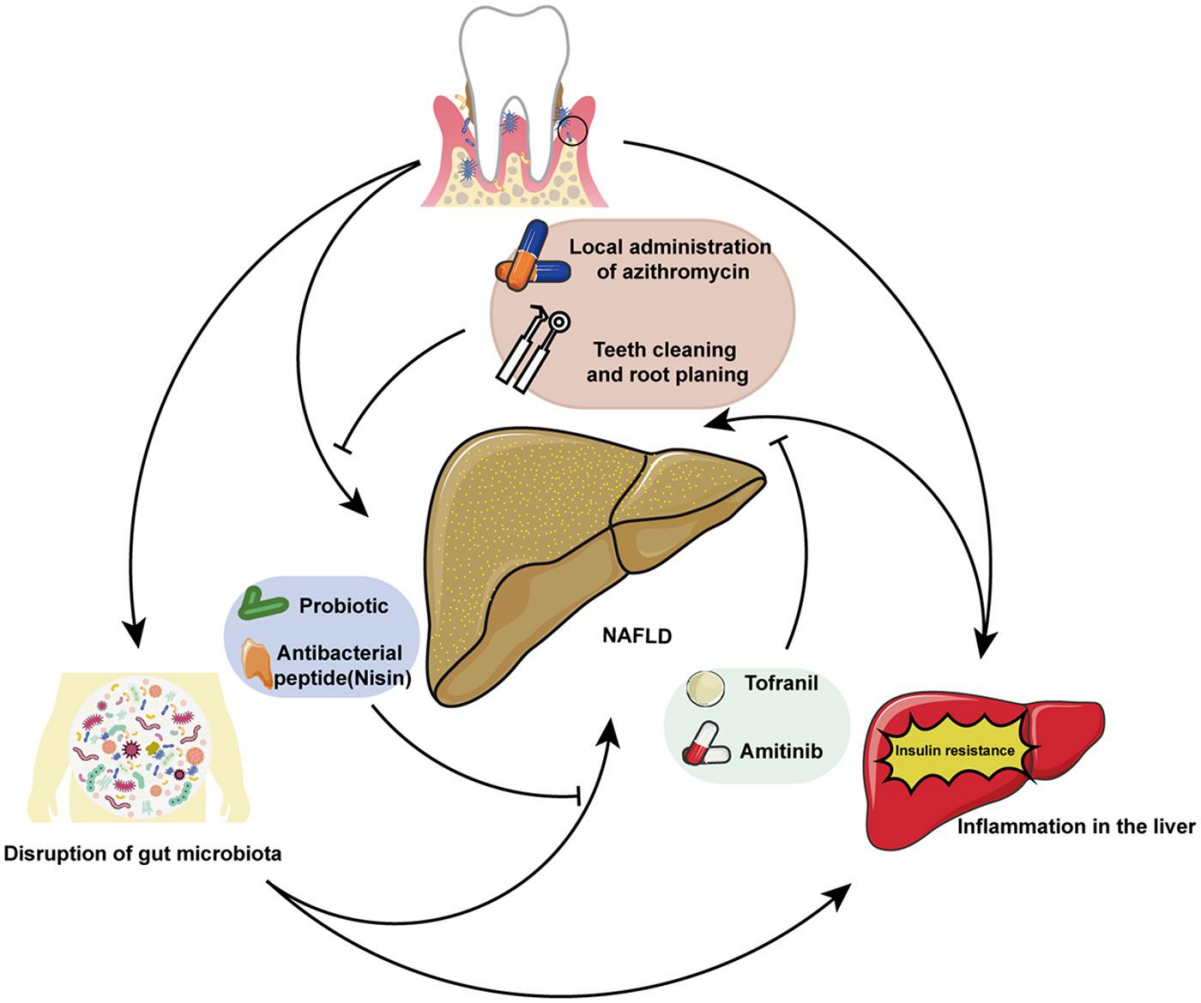
The impact of periodontitis treatment on the progression of NAFLD. Periodontitis treatment, including local administration of azithromycin and mechanical teeth cleaning with root planing, reduces oral inflammation and bacterial load, thereby interrupting the oral–gut–liver axis. This intervention alleviates systemic inflammation and insulin resistance in the liver, which are key drivers of NAFLD. Additionally, modulating gut microbiota through probiotics or antibacterial peptides (e.g., nisin) helps restore microbial balance and gut barrier integrity, further reducing endotoxin translocation and hepatic inflammation. Pharmacological agents such as tofranil and amitinib may also contribute to ameliorating liver pathology by targeting inflammatory pathways. Together, these strategies demonstrate that effective periodontal therapy can positively influence liver health and slow the progression of NAFLD.

Accumulating evidence from both clinical and basic research demonstrates that non-surgical periodontal therapy can modulate the progression of NAFLD and its more severe form, nonalcoholic steatohepatitis (NASH). Key non-surgical periodontal therapy modalities with documented effects on NAFLD include: Anti-inflammatory therapy: Particularly involving antibiotics (e.g., azithromycin, clindamycin) (50, 53). Acid sphingomyelinase (ASMase) inhibition: Using agents like imipramine hydrochloride (51). Microbiome-targeted therapies: Designed around the oral-gut-liver axis, such as probiotics (e.g., Lactobacillus spp.), prebiotics, bacteriocins, and synbiotics. Periodontal debridement: Scaling and root planing (SRP) and oral hygiene reinforcement. These interventions may exercise beneficial effects on periodontitis and NAFLD/liver fibrosis independently, concurrently, or through cross-talk between the two conditions (Figure 3).

### 3.1 Antibiotic therapies and their role in modulating inflammation (e.g., Azithromycin, Amitriptyline)

Anti-inflammatory approaches, especially antibiotic regimens, represent the most widely investigated strategy with dual efficacy against periodontitis and NAFLD. For instance, topical application of 1% amitriptyline gel combined with amitriptyline mouthwash ameliorated periodontitis, as evidenced by significant reductions in salivary levels of TNF-α, PGE2, and nitric oxide (*p* ≤ 0.001) (57). Azithromycin, administered locally or systemically to eradicate *P. gingivalis* infection, demonstrated potent therapeutic effects against odontogenic infection–driven non-alcoholic steatohepatitis (NASH) in a mouse model of high-fat diet–induced NASH with concurrent *P. gingivalis* infection. In this model, treatment significantly attenuated key pathological features of NASH: it reduced hepatic expression of pro-inflammatory cytokines (TNF-α, IL-1β), suppressed fibrosis mediators (galectin-3, pSmad2), decreased the formation of hepatic crown-like structures (hCLSs), and lowered both the NAFLD activity score (NAS) and fibrosis area, collectively inhibiting NASH progression (53).

### 3.2 Targeting ASMase with imipramine

Lu Z, Chowdhury N, et al. investigated the mechanistic link whereby periodontitis exacerbates NAFLD/NASH, identifying increased insulin resistance and hepatic inflammation as key drivers (51). Imipramine hydrochloride, an ASMase inhibitor, effectively mitigated these effects: reducing insulin resistance and liver inflammation, and consequently improving both NASH and periodontitis. These findings highlight the therapeutic potential of ASMase inhibition in attenuating the adverse impact of periodontitis on NAFLD progression.

### 3.3 Microbiome-targeted therapies (probiotics, prebiotics, bacteriocins)

Given the potential cascade from oral dysbiosis (dominated by pathobionts/periodontal pathogens) to gut dysbiosis and subsequent NAFLD, microbiome-modulating strategies hold promise for prevention and management (58, 59). Clinical evidence supports this: a randomized controlled trial in NAFLD patients found that a synbiotic (probiotic blend similar to VSL3 plus fructooligosaccharides) administered twice daily for 28 weeks significantly improved liver function, inflammation, and fibrosis markers compared to placebo (60). A systematic meta-analysis of 21 RCTs further concluded that probiotic or synbiotic supplementation effectively improves liver function, stiffness, and serum biomarkers of liver injury in NAFLD patients (61).

Among probiotics, Lactobacillus spp. are the most extensively studied. They demonstrate efficacy against oral and systemic microbial dysbiosis (62) and can combat hepatic steatosis by reducing intestinal fatty acid absorption (63). Specific probiotic strains show broader benefits: Bifidobacterium animalis subsp. lactis HN019 (PROB) significantly reduced alveolar bone loss in periodontitis models. Concurrently, it improved gut morphology (increased villus height, deeper crypt depth) and mitigated periodontitis-induced sequelae like hepatic lipogenic gene upregulation and steatosis (64). Nisin, an antimicrobial peptide (bacteriocin) produced by Lactococcus lactis, reshaped the oral, gut, and liver microbiomes towards a healthier composition/diversity in infected mice. Critically, Nisin treatment reduced hepatic lipid deposition and oxidative stress (lower malondialdehyde, MDA), thereby alleviating hepatocyte damage (65). Probiotics generally promote a restorative proliferation of a healthy microbiome while dampening inflammation (66). Although research specifically on microbiome-targeted therapy for periodontitis-associated NAFLD remains limited, the use of probiotics, prebiotics, and bacteriocins constitutes a promising therapeutic avenue for comorbid patients.

### 3.4 Other non-surgical interventions

Oral Hygiene Improvement: A large Korean national study (*n* = 6,352 adults) revealed an inverse association between toothbrushing frequency and NAFLD risk. Brushing ≥3 times/day was associated with a 44% lower risk (adjusted OR = 0.56) compared to brushing ≤1 time/day. Notably, this protective effect was most pronounced, and the risk most elevated with infrequent brushing (≤1 time/day), among high-risk subgroups like smokers (OR = 2.26) and diabetics (OR = 4.52) (67). Professional Periodontal Debridement (Scaling and Root Planing—SRP): SRP effectively counteracted NAFLD progression in experimental models. Treatment downregulated hepatic CD36 (a fatty acid transporter) at mRNA and protein levels, reduced intrahepatic fat accumulation, improved insulin resistance, and lowered serum C-reactive protein (CRP), thereby mitigating the systemic inflammation induced by periodontitis (34). Adjunctive Anti-inflammatory/Antioxidant Agents: Bromelain (derived from Ananas comosus) significantly improved periodontitis (reduced Gingival Bleeding Index [GBI], tooth mobility [TM], probing pocket depth [PPD]) and associated fatty liver disease. Its benefits encompassed reduced oral oxidative stress (↓MDA, ↑GSH), improved systemic dyslipidemia (↓cholesterol, ↓triglycerides), and attenuated liver injury (↓ALT, ↓AST), attributed to its anti-inflammatory and antioxidant properties (68). Conclusion on Non-Surgical Management: Enhanced periodontal health management—encompassing improved oral hygiene practices (increased brushing frequency), regular professional debridement (SRP), and adjunctive anti-inflammatory/antioxidant therapies—represents a valuable non-pharmacological strategy for potentially delaying the progression of NAFLD/NASH.

However, the translation of these promising findings into clinical practice requires careful consideration of the evidence level and associated challenges. The evidence for antibiotic efficacy derives primarily from controlled animal models of NASH, representing a moderate level of preclinical evidence but necessitating validation in human trials. A major clinical concern is the risk of promoting antibiotic resistance, which limits the feasibility of long-term or widespread antibiotic use for a chronic condition like NAFLD. There is a notable lack of randomized controlled trials (RCTs) directly evaluating probiotics in patients with periodontitis-associated NAFLD, making the current evidence preliminary. In contrast, non-surgical periodontal therapy (scaling and root planing) and improved oral hygiene are supported by clinical observational data and some interventional studies, offering a higher level of practical evidence with a favorable risk-benefit profile. Future research should prioritize well-designed human RCTs to establish causal efficacy, explore non-antibiotic anti-inflammatory strategies, and define precise microbial or host-targeted interventions for this comorbid condition.

## 4 Conclusion

In summary, periodontal disease has been established as a risk factor associated with multiple systemic conditions, underscoring the systemic implications of localized periodontal inflammation. Concerning the mechanistic link between periodontitis and NAFLD, a key biological pathway involves the low-grade chronic inflammation characteristic of periodontitis propagating systemically. This systemic inflammation is a critical driver of insulin resistance (IR), and the interplay between chronic inflammation and IR synergistically promotes the development and progression of NAFLD (69). Periodontal pathogens, notably *P. gingivalis*, contribute to IR via the release of LPS and pro-inflammatory cytokines (70). Supporting the role of specific pathogens, murine studies have provided the inaugural comprehensive assessment of hepatic gene expression profiles and gut microbiota alterations following exposure to *A. actinomycetemcomitans* (71). However, a notable gap exists in basic research investigating NAFLD induction by another key periodontal pathogen, *F. nucleatum*.

Critically, non-surgical periodontal therapy (NSPT) demonstrates potential in modulating the progression of MAFLD (Metabolic dysfunction-Associated Fatty Liver Disease) and its more severe form, NASH. While periodontitis itself is manageable through established systemic periodontal treatment protocols, the specific application of anti-periodontitis pharmacotherapies as adjunctive treatments for MAFLD has not yet gained widespread clinical traction. Consequently, this review has synthesized current evidence elucidating the pathogenic mechanisms linking periodontitis to fatty liver disease. This synthesis aims to provide a compelling justification for exploring targeted anti-periodontitis interventions as a novel therapeutic approach for MAFLD.

Despite the accumulating evidence reviewed herein supporting an association between periodontitis/periodontal pathogens and MAFLD, several limitations warrant careful consideration. A significant challenge lies in the substantial methodological heterogeneity across the included studies. Key sources of heterogeneity encompass variations in ethnicity, diagnostic criteria applied for both periodontitis and NAFLD/MAFLD, and the historical context (time periods) of the investigations. To strengthen the validity and generalizability of the proposed association, future research must prioritize comprehensive, well-designed, large-scale prospective clinical studies and rigorous meta-analyses specifically aimed at minimizing heterogeneity. Such high-quality evidence is essential to definitively establish causality and inform clinical practice.

## Data Availability

The original contributions presented in the study are included in the article/supplementary material, further inquiries can be directed to the corresponding author/s.
